# Classical Hodgkin Lymphoma on the Background of Castleman Disease: A Case Report

**DOI:** 10.7759/cureus.44930

**Published:** 2023-09-08

**Authors:** Mohammad Ma'koseh, Akram Al-Ibraheem, Nidal Almasri, Eman Hamed, Kamal Alrabi

**Affiliations:** 1 Medical Oncology, King Hussein Cancer Center, Amman, JOR; 2 Nuclear Medicine, King Hussein Cancer Center, Amman, JOR; 3 Pathology, King Hussein Cancer Center, Amman, JOR; 4 Internal Medicine, Faculty of Medicine, Jordan University Hospital, Amman, JOR

**Keywords:** bulky, chemotherapy, unicentric, castleman disease, hodgkin lymphoma

## Abstract

Castleman disease (CD) is a rare lymphoproliferative disorder that is associated with an increased risk for lymphoma. The association between CD and classical Hodgkin lymphoma (HL) is rare. The patient described here is a 44-year-old, HIV-seronegative male who presented with significant weight loss, fever, night sweats, and right axillary swelling. Imaging showed bulky infraclavicular, subpectoral, and axillary lymph nodes. A biopsy revealed classical HL on the background of a human herpesvirus-8 (HHV-8)-negative plasma cell variant of CD. The patient had a complete remission after six cycles of doxorubicin, bleomycin, vincristine, and dacarbazine (ABVD) that were followed by consolidative radiotherapy and continued to be disease-free for more than two years.

## Introduction

Castleman disease (CD), also known as angiofollicular lymph node hyperplasia, is a heterogeneous group of lymphoproliferative disorders with variable clinical presentations that share a spectrum of histopathological features. It was first described in 1956 as "lymph node enlargement with pathological features of an increased number of lymphoid follicles, germinal center involution, and marked capillary proliferation" [1].

There are three histopathological subtypes of CD: hyaline vascular (HV), plasma cell variant (PC), and mixed [2]. The three clinical subtypes of CD, the unicentric CD (UCD), idiopathic multicentric CD, and Kaposi's sarcoma herpesvirus/human herpesvirus 8 (KSHV/HHV8)-associated multicentric CD, were identified in the most recent World Health Organization (WHO) classification of lymphoid neoplasms as tumor-like lesions with B-cell predominance [3].

Secondary malignancies are not uncommon in CD. UCD is associated with an increased risk of dendritic cell sarcoma [4], classical Hodgkin lymphoma (cHL), and non-Hodgkin lymphoma (NHL) [5,6]. UCD and HL usually coexist in the same lymph node and is of the PC variant, while NHL is usually found in a distant anatomical site and is of the HV subtype [7]. In a recent review by Lyapichev et al., a total of 37 cases of CD and cHL were reported in the literature [8]. We report a case of UCD with cHL coexistent in the same lymph node that had a sustained remission following treatment with the standard cHL chemotherapy and consolidative radiotherapy.

## Case presentation

A 44-year-old male presented with one-year history of weight loss, anorexia, fever, night sweats, and an enlarging right axillary mass. There was no history of skin rash, neuropathic pain, limb weakness, or seizures. On physical examination, there were multiple enlarged right axillary lymph nodes measuring about 7 to 8 cm. Chest examination was clear with no crackles or signs of volume overload. 

Initial blood tests were remarkable for a hemoglobin of 6.8 grams/dl, a white blood cell count of 11.4x109/L, a lymphocyte count of 0.8x109/L (7.6%), an erythrocyte sedimentation rate (ESR) of >150 mm/hour, an albumin of 1.77 grams/dl, and a seronegative human immunodeficiency virus (HIV) test. Initial blood tests are detailed in Table 1. 

**Table 1:** Initial blood tests.

Blood test	Value	Normal range
White blood cells	11.4	4-11
Neutrophil count (%)	9 (79%)	2.2-7.1 (40-65%)
Lymphocyte count (%)	0.8 (7.5%)	0.9 -3.4 (25-40%)
Monocyte count (%)	1.4 (12.1%)	0.2-0.8 (2-8%)
Hemoglobin (g/dl)	6.7	13-16.6
Platelet count	415	150-400
Erythrocyte sedimentation rate (mm/hour)	>150	0-15
C-reactive protein (mg/dl)	60.5	< 8
Beta-2 microglobulin (mg/L)	5.2	0.8-2.2
Serum creatinine (mg/dl)	0.5	0.5-1.12
Serum total protein (g/dl)	8.89	6.2-8.2
Albumin (g/dl)	1.17	4-5.1
Lactate dehydrogenase (U/L)	138	122-222
Serum iron (mcg/dl)	37	50-150
Total iron binding capacity (mcg/dl)	128	250-400
Transferrin saturation (%)	29%	20-50%
Direct coomb’s test	Negative	Negative
Fibrinogen (mg/dl)	1000	200-400

**Figure 1:**
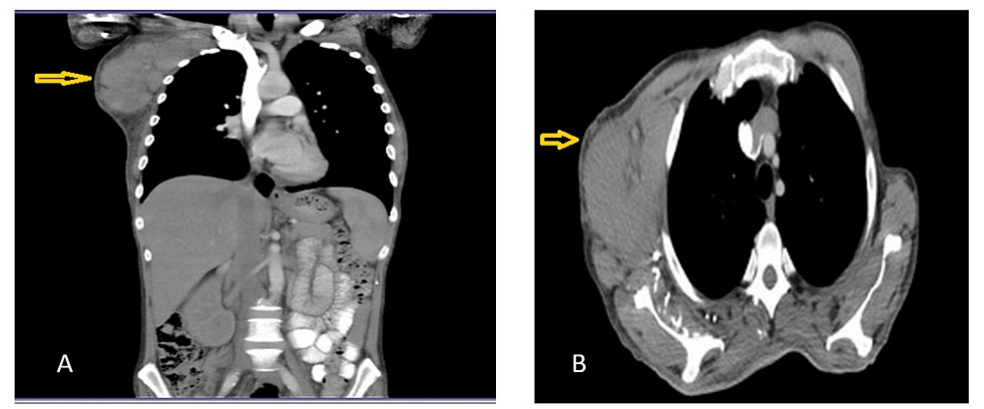
CT scan showing enlarged axillary and subpectoral lymph nodes on the coronal view (A) and transverse view (B).

A computed tomography (CT) scan showed multiple large oval-shaped soft tissue masses in the right supra and infraclavicular regions that extended along the whole right axillary and subpectoral regions. The largest lymph node measured 11x6 cm and showed areas of central necrosis. The masses were closely related to the anterior right ribs (Figure 1). 

**Figure 2:**
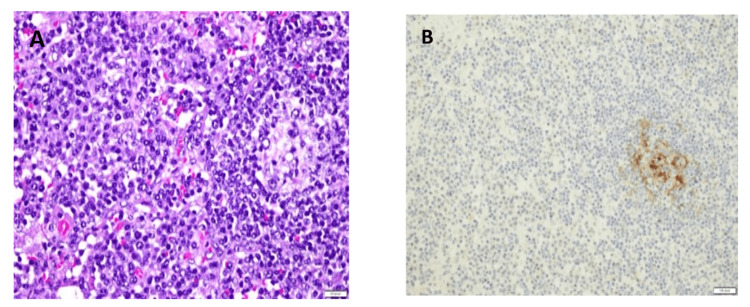
(A) Microscopic section of the portion of the lymph node involved by Castleman disease, showing regressed germinal center at the left side surrounded by mantle cells. Most of the cells outside the regressed follicle are sheets of plasma cells, which were polyclonal. (B) CD21 immunostains of the area shown in A, demonstrating that the cells in the center of the follicle are follicular dendritic cells, which appear brown in this stain.

A trucut biopsy of the axillary lymph node was taken. Histologic examination showed enlarged lymph nodes with a relatively preserved architecture. Many lymphoid follicles were seen throughout the nodes with variable-sized germinal centers ranging from hyperplastic to atrophic. The interfollicular areas were infiltrated by sheets of plasma cells (Figure 2). 

**Figure 3:**
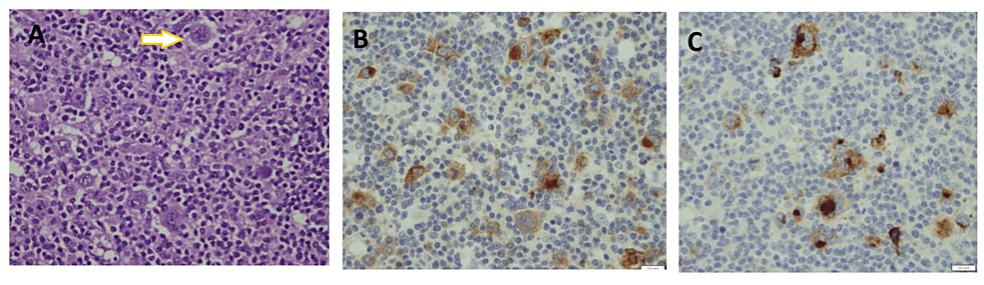
(A) In other parts of the lymph node, classic Reed-Sternberg (RS) cells with binucleated and multilobed nuclei surrounded mostly by sheets of small lymphocytes and plasma cells. (B) RS cells are CD30 positive as highlighted by the brown stain. (C) RS cells are CD15 positive as highlighted by the brown stain.

The architecture appeared to be disturbed and infiltrated by polymorphic cells, including Reed-Sternberg (RS) cells (Figure 3A). The RS cells were positive for CD30 and CD15 (Figures 3B, 3C) but negative for CD20, CD3, CD45, and CD21. CD21 immunostain decorated the follicular dendritic cells within the germinal centers. Kappa and lambda light chain immunostains confirmed the polyclonal nature of the plasma cells. HHV-8 immunostaining was negative. 

**Figure 4:**
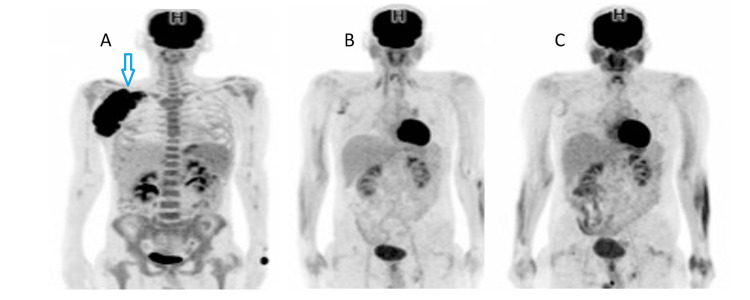
(A) A maximum intensity projection (MIP) PET scan at baseline demonstrates an intensely hypermetabolic conglomerate lymphadenopathy in the right axillary subpectoral region. (B) The MIP PET scan after two cycles of ABVD demonstrates a partial metabolic response with a dramatic regression of the intensely hypermetabolic lymphadenopathy but with mildly increased residual FDG uptake (Deauville 5PS=4). (C) The MIP PET scan after four cycles of ABVD demonstrates a complete metabolic response with minimal residual nonspecific FDG metabolic activity (Deauville 5PS=2). MIP: maximum intensity projection, PET: positron emission tomography, ABVD: doxorubicin, bleomycin, vincristine, and dacarbazine, Deauville 5PS: Deauville five-point scale, FDG: fludeoxyglucose F18

A positron-emission computed tomography (PET-CT) scan showed multiple enlarged hypermetabolic right-sided infraclavicular, axillary, and subpectoral lymph nodes, the largest of which measured about 13 cm with SUVmax = 26 (Figure 4A). The bone marrow biopsy was normal.

Accordingly, the patient was diagnosed with unfavorbale stage II cHL on the background of CD. He was treated with six cycles of ABVD (doxorubicin, bleomycin, vincristine, and dacarbazine). After the first cycle of ABVD, there was a marked improvement in systemic symptoms and a regression in the size of the axillary mass. A PET-CT after the second cycle showed a significant metabolic and size regression of the hypermetabolic lymph nodes (Deauville five-point scale (Deauville 5PS)=4) (Figure 4B). After the fourth and sixth cycles, the PET-CT revealed a complete metabolic response (Deauville 5PS=2) (Figure 4C). Consolidative involved-site radiotherapy (30 Gy in 17 fractions) was given and finished in July 2020. He was continued on follow-up with clinical examination and a CT scan every six months with no evidence of relapse for more than two years. 

## Discussion

We report a rare a case of HIV-negative interfollicular cHL coexisting with an HHV8-negative PC-type UCD in a patient with an unfavorable bulky stage IIB disease who was successfully treated with standard cHL chemotherapy and radiotherapy.

The relationship between cHL and UCD, although rare, has been well documented. There is no clear explanation for this association. Interleukin-6 (IL-6) has been implicated in the pathogenesis of cHL and CD [9,10]. Furthermore, RS has been shown to produce IL-6, as evidenced by being positive for IL-6 and the fact that a higher IL-6 level can be used to predict relapse [8,11,12].

The presentation of our patient with systemic symptoms is not unusual, as about one half of patients with CD coexistent with cHL were reported to have B symptoms [13]. However, only 11% of patients were reported to have involvement of the axillary lymph node [8].

Missing the diagnosis of cHL in CD is not uncommon [7,14]. One study reported that the diagnosis was delayed in 13 out of 19 cases [15]. This may be caused by the abundant inflammatory changes observed in CD, causing the pathologist to miss the presence of RS cells [14]. Meanwhile, plasmacytic histological changes may represent reactive changes secondary to cHL [7]. Although an excisional lymph node biopsy is usually recommended, a tru-cut needle biopsy was enough to diagnose both diseases in our case.

The interfollicular (IF) variant of cHL, the pathological subtype reported in our patient, accounts for about 50-60% of cHL coexisting with CD [8,13]. Lukes first identified the IF variant of cHL in 1971 [16]. It is distinguished by lymph node involvement, the paracortical and intrafollicular distribution of hyperplastic lymphoid follicles, and a lack of RS cells [17]. Although IF cHL is not recognized as a separate entity, it was categorized as a growth pattern in mixed cellularity cHL [17,18]. As a result, some of the cases of mixed cellularity and possibly lymphocyte-rich cHL reported to be associated with CD may have an IF growth pattern, which may have resulted in an underestimation of its real frequency in this context [8].

Despite the fact the PC histology represents 10-25% of UCD [17,18], it is found in about 85-90% of cHL associated with CD [8,13]. This may be related to the production of IL-6 and other cytokines by the RS cells and germinal centers [7]. Similarly, HIV and HHV-8 negativity was previously reported in most cases [4,8,19].

The treatment of cHL on the background of CD usually follows the standard approach for the treatment of cHL [20]. Our patient had a sustained, complete response to ABVD and radiotherapy despite having unfavorable features, including the B symptom, bulky disease, a high ESR, lymphopenia, and hypoalbuminemia.

## Conclusions

Patients with HHV-negative PC-UCD should be evaluated for the coexistence of HL. The diagnosis can be missed or delayed in a significant proportion of patients. Early diagnosis and administration of proper treatment may result in curing both conditions.
